# The selective ERβ agonist AC 186 reduces polyinosinic:polycytidylic acid (poly I:C)-induced inflammatory responses in BEAS-2B lung epithelial cells

**DOI:** 10.3389/fphar.2025.1706638

**Published:** 2025-10-21

**Authors:** Olumayokun A. Olajide, Hope A. Ogiogio

**Affiliations:** Department of Pharmacy, School of Applied Sciences, University of Huddersfield, Huddersfield, United Kingdom

**Keywords:** AC186, bronchial epithelial cells, inflammation, NF-κB, NLRP3 inflammasome, ERβ

## Abstract

**Background:**

Acute respiratory distress syndrome (ARDS) is characterised by inflammation with the accompanying release of pro-inflammatory cytokines. AC186 is an oestrogen receptor agonist, which has shown anti-inflammatory activity. This study investigated effects of AC186 on poly I:C-induced inflammation in human bronchial epithelial (BEAS-2B) cells.

**Methods:**

Supernatants from poly I:C-stimulated BEAS-2B cells treated with AC186 (1.25, 2.5 and 5 µM) were analysed for levels of tumour necrosis factor-alpha (TNFα), interleukins-6 (IL-6), -1β (IL-1β) and −8 (IL-8), using ELISA. Protein expression of phospho-p65 NF-κB was evaluated using Lumit^®^ Immunoassay, while nuclear localisation, DNA binding and transcriptional activity of NF-κB were evaluated using immunofluorescence, transcription factor ELISA and reporter gene assays, respectively. In cell ELISAS were used to determine effects on NLRP3 and caspase-1 proteins while Caspase-Glo^®^ 1 inflammasome assay was used to determine whether AC186 influenced caspase-1 activity. Experiments were conducted to evaluate effects on ATP production and caspase 3/7 activity.

**Results:**

AC186 produced significant (p < 0.05) reduction in elevated levels of TNFα, IL-6, IL-1β and IL-8 in BEAS-2B cells, in comparison with poly I:C stimulation alone. Increased phosphorylation of p65 was significantly (p < 0.01) reduced in the presence of AC186 (2.5 and 5 µM), while nuclear localisation of p65, as well as DNA binding and transactivation were blocked with 2.5 and 5 µM of the compound. AC186 (2.5 and 5 µM) reduced protein levels of both NLRP3 inflammasome and caspase-1, as well as caspase-1 activity. Co-administration of ICI 182780 (10 nM) with AC186 (5 μM) prior to stimulation with poly I:C resulted in higher levels of TNFα and IL-6 secretion, compared to AC186 pre-treatment alone. Following incubation of AC186 (2.5 and 5 μM) with poly I:C-stimulated BEAS-2B cells for 72 h, there was significant improvement in viability as well as reduction in caspase 3/7 activity, in comparison with poly I:C alone.

**Conclusion:**

These results suggest that AC-186 produced anti-inflammatory activity in poly I:C-stimulated BEAS-2 cells, through mechanisms involving inhibition of NF-κB and NLRP3/caspase-1/IL-1β activation. AC186 also protected BEAS-2B cells against poly I:C-mediated apoptosis and death suggesting that this compound have potentials in reducing inflammatory events associated with ARDS caused by viral infections.

## Introduction

Acute lung injury (ALI), also known as moderate acute respiratory distress syndrome (ARDS) (Mowery et al., 2020; Summers et al., 2016; Ziaka and Exadaktylos, 2025) is a high mortality rate lung disease, which is associated with severe lung inflammation (Liu et al., 2025). Despite new developments in the understanding and treatment of ARDS, mortality remains high at 30–40% (Matthay et al., 2019). At the cellular level, ARDS is characterised by impairment of alveolar–capillary membrane integrity, migration of neutrophils, and release of pro-inflammatory cytokines and cytotoxic reactive oxygen species (Mowery et al., 2020).

Respiratory viruses, such as influenza, respiratory syncytial viruses, rhinoviruses and coronaviruses are responsible for infections of the upper or lower respiratory tract, which affect millions of people worldwide (Khomich et al., 2018). Viral infections are linked with the release of excessive amounts of pro-inflammatory and pro-oxidant mediators (including cytokines and chemokines) in the bronchial epithelium, leading to recruitment of immune cells, tissue damage and cell death (Becker and Soukop, 1999; Khomich et al., 2018; Yamaya et al., 2023).

Anti-inflammatory strategies which have been adopted for managing ARDS caused by viral infections include have mainly focused on the anti-inflammatory steroids like dexamethasone. The benefits of dexamethasone in SARS-CoV-2 were highlighted in an article published by the Recovery Collaborative Group et al. (2021). However, the use of dexamethasone has been linked to inhibition of T cells and B cells, with subsequent immunosuppression and other side effects (Meybodi et al., 2024). Consequently, investigations have focused on identifying novel alternative anti-inflammatory interventions for treating ARDS following viral infections.

Studies have shown higher severity and mortality in men than women infected with respiratory viruses such as the SARS-CoV-2 (Pivonello et al., 2021; El Aidaoui et al., 2022). Pivonello et al. further suggested that women could be relatively protected from COVID-19 partly because of a less pronounced systemic inflammation. The anti-inflammatory protection against severe COVID-19 has been linked to the concentration of the female hormone, oestradiol and the expression of oestrogen receptors (Ulhaq et al., 2020). Results of a study published by Lizarazo-Taborda et al. (2025) showed a higher expression of IL-8 and MIP-1β in pre-menopausal women group compared to post-menopausal women and men with COVID-19, suggesting that oestrogen receptors may be valuable pharmacological targets to reduce inflammatory complications associated with viral lung infections, including ARDS.

**FIGURE 1:**
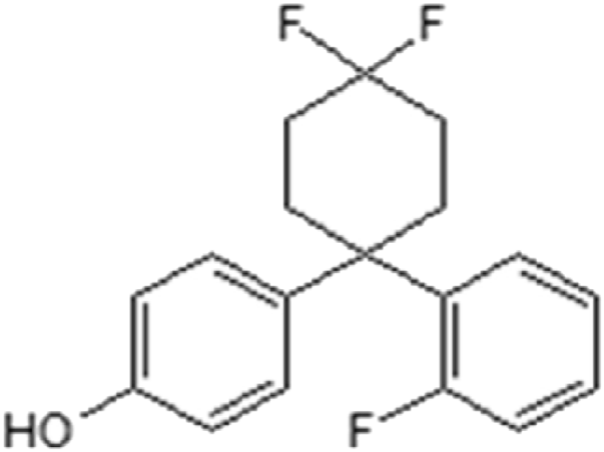
Chemical structure of AC186.

AC186 (4-[4-4-Difluoro-1-(2-fluorophenyl) cyclohexyl] phenol) (Figure 1) is a non-steroidal selective oestrogen receptor modulator, which has been previously shown to produce neuroprotective effects in animal models of Parkinson’s and Alzheimer’s diseases as well as multiple sclerosis (McFarland et al., 2013; George et al., 2013; Itoh et al., 2017) and anti-inflammatory effects in BV-2 microglia activated with bacterial lipopolysaccharide (Katola et al., 2024).

In this study we have investigated the effects of AC186 on inflammation induced by the synthetic viral double-stranded RNA molecule, polyinosinic:polycytidylic acid (poly I:C) in human bronchial epithelial (BEAS-2B) cells.

## Materials and methods

### Materials

AC 186 was purchased from Tocris (United Kingdom). Fibronectin and bovine collagen type I were obtained from Fisher Scientific (United Kingdom), while ICI 182780 (fulvestrant) and bovine serum albumin were purchased from Merck (United Kingdom).

### Cell culture

The human bronchial epithelial (BEAS-2B) cells were purchased from CLS Cell Lines Service GmbH (Germany). The cells were cultured in LHC-9 medium (Gibco) in flasks coated with 0.01 mg/mL fibronectin, 0.03 mg/mL bovine collagen type I and 0.01 mg/mL bovine serum albumin (BSA) dissolved in LHC basal medium (Gibco). Cells were maintained in a 5% CO_2_ incubator at 37 °C.

### Cell viability assay

BEAS-2B cells were seeded out in a fibronectin/bovine collagen type I/bovine serum albumin-coated 96-well plate at 1 x 10^4^ cells/mL and incubated until 70% confluent. Cells were then treated with AC-186 (1.25, 2.5 and 5 µM), while control cells were treated with an equal volume of DMSO. One hour later, cells were stimulated with poly I:C (30 μg/mL) for a further 24 h. Thereafter, 100 µL medium was removed from all wells, followed by addition of 10 μL of 3-(4,5-dimethylthiazol-2-yl)-5-(3-carboxymethoxyphenyl)-2-(4-sulfophenyl)-2H-tetrazolium, inner salt (MTS) solution (Promega, United Kingdom). The plate was then incubated at 37 °C for 4 h, followed by measurement of absorbance at 490 nm with an Infinite™ M200 PRO microplate reader (Tecan, Austria).

### Secretion of pro-inflammatory cytokines and chemokine

Cultured BEAS-2B cells were treated with AC-186 (1.25, 2.5 and 5 µM). One hour later, they were stimulated with poly I:C (30 μg/mL) for a further 24 h. Thereafter, culture supernatants were collected and analysed for levels of TNFα, IL-1β, IL-6, and IL-8, using human ELISA kits (ThermoFisher Scientific). Absorbance values were measured using a Tecan Infinite™ M200 PRO microplate reader (Tecan, Austria), while concentrations of mediators in supernatants were calculated from standard curves.

### Lumit^®^ immunoassay for phospho-NF-κB p65 (Ser536)

The Lumit^®^ immunoassay (Promega, United Kingdom) is a homogeneous cell-based assay (Brian Hwang et al., 2023), which can detect phosphorylation of p65 NF-κB in unmodified cells using immunodetection combined with protein-sub-unit complementation.

BEAS-2B cells were treated with AC-186 (1.25, 2.5 and 5 µM) for 60 min prior to stimulation with poly I:C (30 μg/mL) for a further 60 min. At the end of the experiment, cells were lysed, followed by the addition of an antibody mix containing mouse anti-phospho-p65 antibody S536 (150 ng/mL; Cell Signalling Technology), rabbit anti-p65 antibody (150 ng/mL; Cell Signalling Technology, United Kingdom), Lumit^®^ anti-mouse Ab-LgBiT/SmBiT (3 μL), Lumit^®^ anti-rabbit Ab-SmBiT/LgBiT (3 μL), and immunoassay reaction buffer. The plate was then incubated at 23 °C for 90 min, followed by the addition of Lumit detection reagent. Luminescence was measured with an Infinite™ M200 PRO microplate reader (Tecan, Austria).

### NF-κB transcription factor assay

Effect of AC186 treatment on poly I:C-induced DNA binding of NF-κB was assessed using the NF-κB p65 transcription factor assay (Olajide et al., 2021a). BEAS-2B cells were seeded in a 6-well plate at a density of 1 × 10^4^ cells/mL, followed by incubation with AC186 (1.25, 2.5 and 5 µM) for 60 min, then stimulation with poly I:C (30 μg/mL) for a further 60 min. Thereafter, nuclear extracts were prepared from the cells and subjected to NF-κB DNA binding assay with a transcription factor assay kit (Abcam), according to the manufacturer’s instructions.

### Transient transfection and NF-κB reporter gene assay

BEAS-2B cells were seeded in 24-well plate at a density of 4 × 10^4^ cells/mL until 60% confluence. Thereafter, culture media were replaced with Opti-MEM, with a further incubation for 2 h at 37 °C. Cells were transfected using magnetofectamine O2 transfection reagent (OZ Biosciences) and Cignal NF-κB luciferase reporter (Qiagen) complex, at a ratio 3:1 in 50 µL Opti-MEM (Olajide et al., 2021b). The plate was placed on a magnetic plate (OZ Biosciences) and incubated at 37 °C for 30 min, followed by a further magnetic plate-free incubation for 18 h. Thereafter, medium was changed to LHC-9 medium and cells treated with AC186 (1.25, 2.5 and 5 µM) for 1 h prior to stimulation with poly I:C (30 μg/mL) for a further 4 h. This was followed by a Dual-Glo^®^ reporter assay (Promega). Firefly and renilla luminescence intensities were measured using an Infinite™ M200 PRO microplate reader (Tecan, Austria).

### Confocal microscopy for nuclear translocation of NF-κB

BEAS-2B cells were seeded into 12 mm Nunc glass base dishes (ThermoFisher Scientific) at a density of 5 x 10^4^ cells/mL. Cells were treated with AC186 (1.25, 2.5 and 5 µM) for 1 h and then stimulated with poly I:C (30 μg/mL). After 24 h, cells were washed twice with phosphate buffered saline (PBS) and fixed with 4% formaldehyde for 10 min at room temperature. They were then washed twice with PBS and blocked with UltraCruz^®^ blocking reagent (Santa Cruz Biotechnology) for 60 min at room temperature. Afterwards, cells were washed and incubated with NF-κB p65 Alexa Fluor^Ò^ 488 antibody (Santa Cruz; 1:250) overnight at 4 °C. Thereafter, cells were washed with PBS and counterstained with DAPI for 1 min. Fluorescence images were acquired using a Zeiss LSM 880 confocal microscope.

### Caspase-Glo inflammasome assay

The Caspase-Glo^®^1 inflammasome assay kit (Promega) (Olajide et al., 2022) was used to measure the activity of caspase-1 directly in live cells BEAS-2B cells, which were treated with AC186 (1.25, 2.5 and 5 µM), prior to stimulation with poly I:C (30 μg/mL) for 24 h. Culture supernatants were collected and mixed with an equal volume of Caspase-Glo^®^ 1 reagent in a 96-well plate. The contents of the wells were mixed using a plate shaker at 400 rpm for 30 s. The plate was then incubated at room temperature for 60 min, followed by luminescent measurement of caspase-1 activity with an Infinite™ M200 PRO microplate reader (Tecan, Austria).

### In cell western ELISA for NLRP3 and caspase-1

The in cell western ELISA is a widely used alternative to traditional Western blotting because it allows the rapid quantification of proteins in fixed cells (Olajide et al., 2021a). BEAS-2B cells in 96-well plates were treated with AC186 (1.25, 2.5 and 5 µM), prior to stimulation with poly I:C (30 μg/mL) for 24 h. Cells were then fixed with 8% paraformaldehyde solution for 15 min, followed by overnight incubation with rabbit anti-NLRP3 (Cell Signalling Technology; #15101) and rabbit anti-caspase-1 (Cell Signalling Technology; #83383) antibodies at 4 °C. Thereafter, cells were incubated with anti-rabbit HRP secondary antibody for 2 h at room temperature. This was followed by the addition of 100 μL avidin HRP substrate and absorbance measured at 450 nm with a Tecan Infinite M Nano microplate reader. Readings were normalised with Janus Green normalisation stain (Abcam).

### Effects of ICI 182780 on the anti-inflammatory effects of AC186

Cultured BEAS-2B cells were treated with either AC-186 (5 µM) or ICI 182780 (10 nM) + AC186 (5 µM). One hour later, they were stimulated with poly I:C (30 μg/mL) for a further 24 h. Culture supernatants were analysed for levels of TNFα and IL-6.

### Effect of AC186 on cell death and apoptosis induced by poly I:C

Poly I:C has been shown to induce death and apoptosis in BEAS-2B cells (Koizumi et al., 2016). Therefore, experiments were conducted to determine whether AC186 could prevent poly I:C-induced damage to these cells. To investigate cell death, the number of viable cells in culture was based on quantitation of the adenosine triphosphate (ATP), which is an indicator of metabolically active cells. To achieve this, a CellTiter-Glo^®^ luminescent (ATP) cell viability assay (Promega) was used. Cultured BEAS-2B cells were treated with AC186 (1.25, 2.5 and 5 µM) for 60 min, followed by incubation with poly I:C (30 μg/mL) for 72 h. Thereafter, 100 µL of CellTiter-Glo^®^ reagent were the plate was then shaken on an orbital shaker for 2 min to induce cell lysis. Following incubation at room temperature for 20 min, luminescence signal was read with a Tecan Infinite M Nano microplate reader.

Caspase 3/7 activity was used as a measure of apoptosis and was assessed using the Caspase-Glo^®^ 3/7 assay system (Promega). Cultured BEAS-2B cells were treated as described for viability experiments. At the end of the incubation period, 100 µL of cell culture medium was removed from each well, followed by the addition of 100 µL of Caspase-Glo^®^ reagent (a mixture of Caspase-Glo^®^ 3/7 buffer and substrate). Thereafter, a reaction mixture was achieved by shaking the plate briefly, followed by incubation for 30 min. Luminescence was measured with a Tecan Infinite M Nano microplate reader.

### Statistical analysis

Values are expressed as mean ± SEM for 3 independent experiments. Data were analysed using one-way analysis of variance (ANOVA) followed by post-hoc Tukey’s test. Data from experiments to determine effects of oestrogen receptor antagonist on anti-inflammatory effect of AC186 were analysed using one-way ANOVA followed by post-hoc Dunnett’s test for multiple comparisons. Values with p < 0.05 were statistically significant. Statistical analyses were carried out with GraphPad Prism Software version 10.5.0.

## Results

### Effect of AC186 on the viability of BEAS-2B cells

**FIGURE 2:**
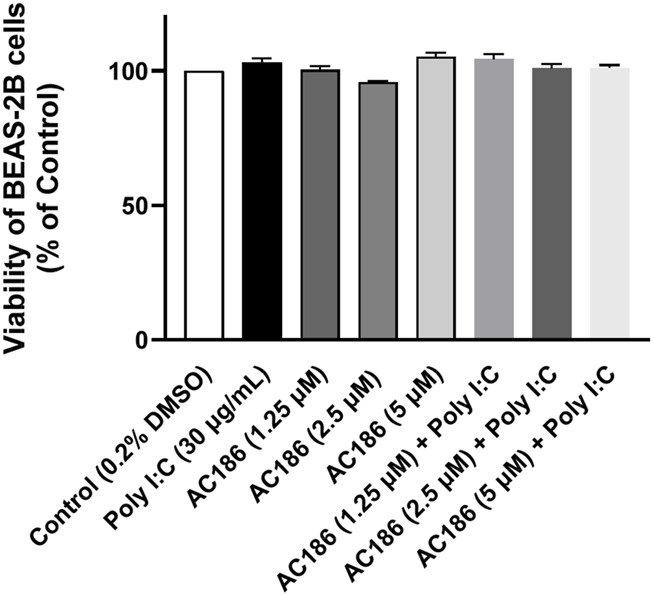
Effect of AC186 on the viability of BEAS-2B cells in the absence and presence of poly I:C. BEAS-2B cells treated with AC186 for 1 h, followed by no stimulation or stimulation with poly I:C (30 μg/mL) for 24 h. Viability was assessed with MTS assay. Results are presented as Mean ± SEM for three independent experiments.

BEAS-2B cells were treated with AC186 (1.25, 2.5 and 5 µM) in the presence of poly I:C to determine whether using these concentrations in subsequent experiments could affect cell viability. Results of MTS viability assays in Figure 2 show that treatments with AC186 alone and in the presence of poly I:C stimulation did not result in significant reduction in the viability of the cells.

### AC186 reduced poly I:C-induced increased release of TNFα, IL-6, IL-1β and IL-8

**FIGURE 3:**
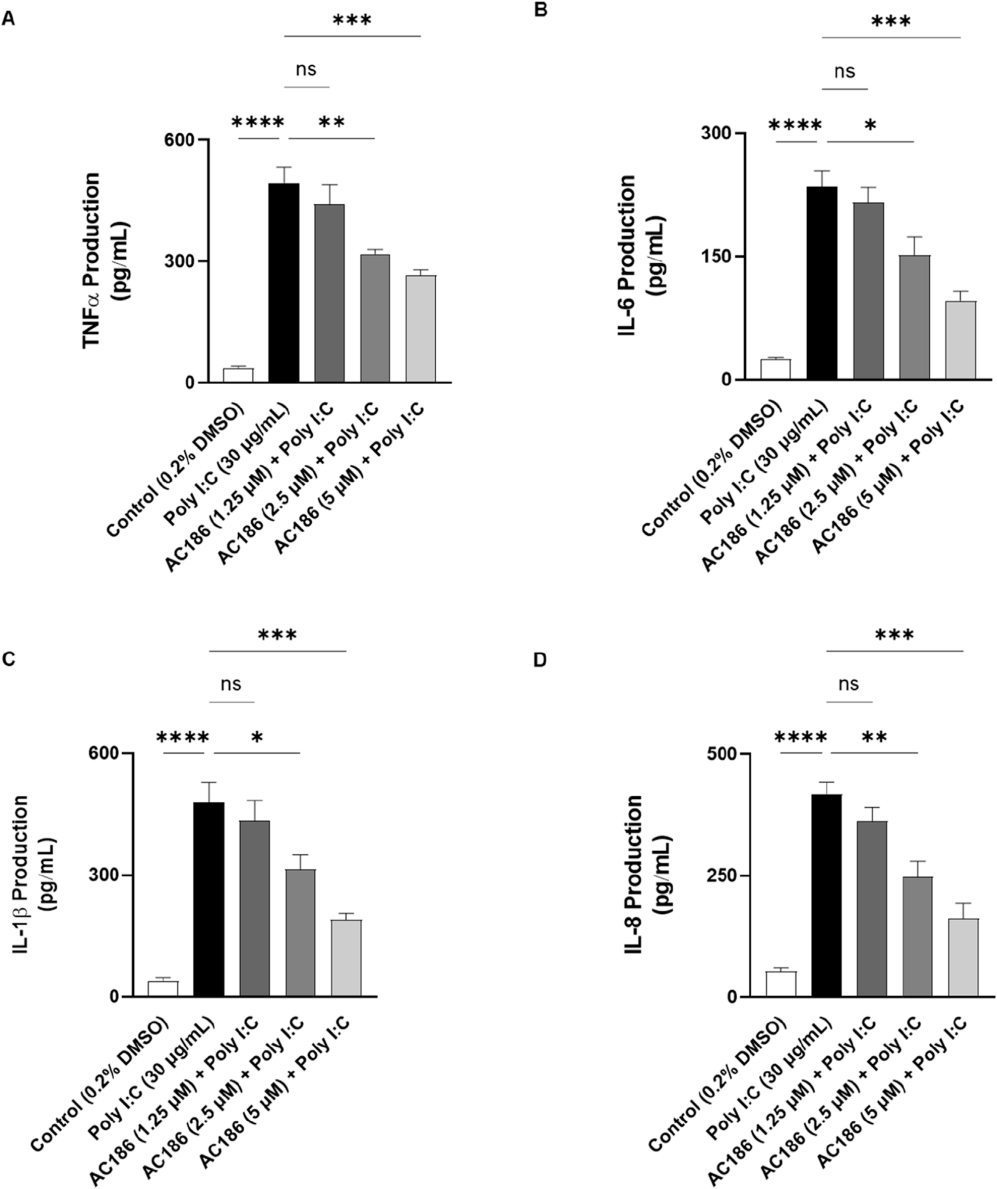
AC186 inhibited the release of inflammatory cytokines in poly I:C-stimulated BEAS-2B cells. Cells were pre-treated with AC186 (1.25, 2.5, and 5 µM) for 1 h prior to poly I:C (30 μg/mL) stimulation for 24 h. Supernatants were analysed for production of TNF-α **(A)**, IL-6 **(B)**, IL-1β **(C)**, and IL-8 **(D)** using Human ELISA kits. Results are presented as mean ± SEM for at least 3 independent experiments; *p < 0.05; **p < 0.01; ***p < 0.001; ****p < 0.0001; one way ANOVA with post-hoc Tukey’s test.

Following stimulation of BEAS-2B cells with poly I:C for 24 h, significant (p < 0.001) increase in TNFα production was detected in culture supernatants (Figure 3A). At a concentration of 1.25 μM, AC186 did not affect elevated levels of TNFα. However, on increasing the concentrations of the compound to 2.5 and 5 μM, significant (p < 0.01) reduction in poly I:C-induced increased TNFα production was observed (Figure 3A). Similarly, poly I:C-induced elevation in the release of IL-6, IL-1β and IL-8 were significantly reduced (p < 0.05) by 2.5 and 5 μM of AC186, while no reductions were observed with 1.25 μM of the compound (Figures 3B–D).

### Effects of AC186 on NF-κB activation in poly I:C-stimulated BEAS-2B cells

**FIGURE 4:**
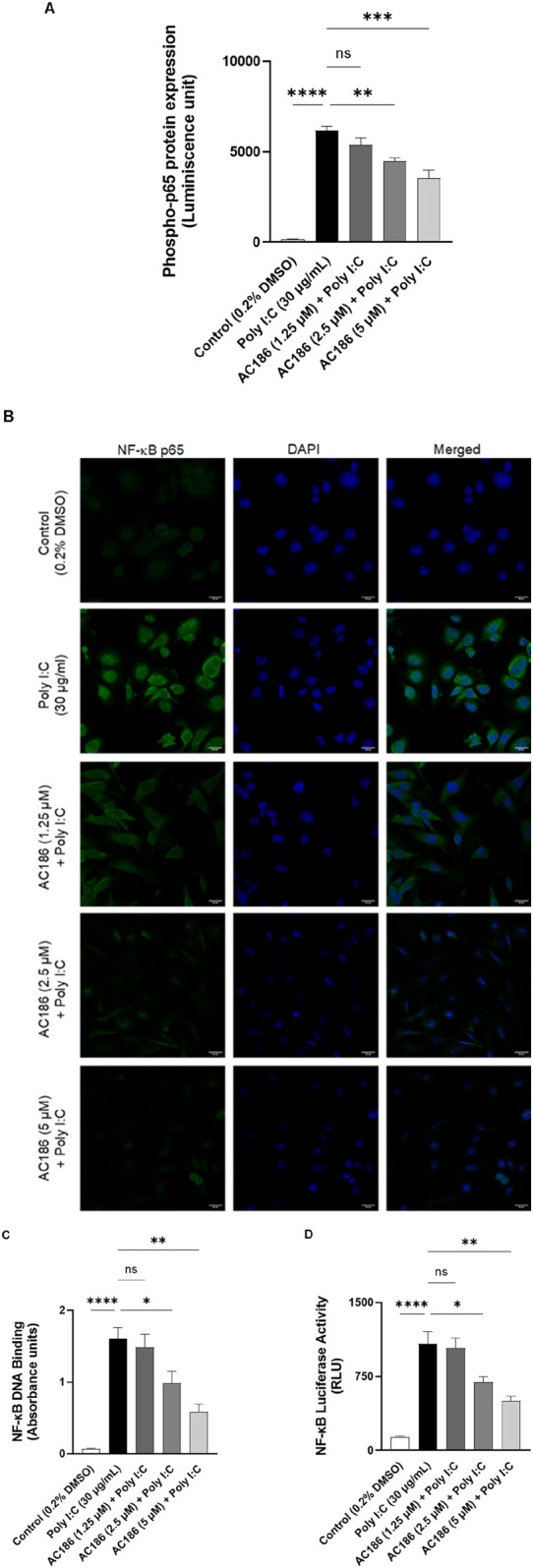
Effects of AC186 on NF-κB activation in poly I:C-stimulated BEAS-2B cells. **(A)** BEAS-2B cells were pre-treated with AC186 (1.25, 2.5, and 5 µM) for 1 h and then stimulated with poly I:C (30 μg/mL). Cell lysates were analysed for phospho-p65 using Lumit^®^ immunoassay (Promega). **(B)** AC186 prevented nuclear localisation of p65 subunit in poly I:C-stimulated BEAS-2B cells. Cells were treated with AC186 (1.25, 2.5, and 5 µM) for 1 h prior to stimulation with poly I:C (30 μg/mL). Nuclear localisation of p65 was detected using immunofluorescence and images acquired with Zeiss LSM 880 confocal microscope. **(C)** AC186 inhibited DNA binding of NF-κB in poly I:C-stimulated BEAS-2B cells. Nuclear extracts were added to 96-well plates to which an oligonucleotide containing the NF-κB consensus site (5′-GGGACTTTCC-3′) had been immobilised, followed by addition of NF-κB and HRP-conjugated antibodies. Absorbance was read in a plate reader at 450 nm. **(D)** AC186 suppressed NF-κB luciferase activity in BEAS-2B cells transfected with NF-κB luciferase plasmid. Results are presented as mean ± SEM for at least 3 independent experiments; *p < 0.05; **p < 0.01; ***p < 0.001; ****p < 0.0001; One way ANOVA with post-hoc Tukey’s test.

Following the observation indicating the anti-inflammatory action of AC186 in poly I:C-stimulated BEAS-2B cells, we investigated whether the effects of the compound were mediated through interference with NF-κB activation. Using the Lumit^®^ immunoassay, protein levels of phsopho-p65 was found to be significantly (p < 0.001) elevated following stimulation of BEAS-2B cells with poly I:C (30 μg/mL). Pre-treatment of cells with the lowest concentration of AC186 (1.25 µM) resulted in insignificant (p < 0.05) reduction in the levels of phospho-p65. However, on increasing the concentrations of AC186 to 2.5 and 5 μM, there were ∼0.7 and 0∼0.6-fold reductions (p < 0.01) in phospho-p65 protein levels, respectively (Figure 4A).

This observation prompted further investigation to determine whether AC186 would prevent nuclear localisation of NF-κBp65. Results of immunofluorescence microscopy in Figure 4B shows that stimulation of BEAS-2B cells with poly I:C caused an increase in nuclear localisation of p65, while pre-treatment with AC186 (1.25, 2.5 and 5 µM) resulted in inhibition of this process. It was further determined whether these effects of AC186 would translate into any modulatory effects on DNA binding and gene regulatory activity of NF-κB. Results of transcription factor assay to determine DNA binding revealed that poly I:C stimulation of BEAS-2B cells resulted in significant (p < 0.001) increase in DNA binding of NF-κB in nuclear extracts. However, in the presence of 2.5 and 5 µM of AC186, significant (p < 0.05) reduction was observed, in comparison with poly I:C stimulation alone (Figure 4C). Reporter gene assays on BEAS-2B cells transfected with the NF-κB plasmid also revealed that the increase in luciferase activity induced by poly I:C was significantly (p < 0.05) reduced when cells were treated with 2.5 and 5 μM, but not 1.25 µM of AC186 (Figure 4D).

### AC186 reduced poly I:C-induced activation of NLRP3/caspase-1 signalling

**FIGURE 5:**
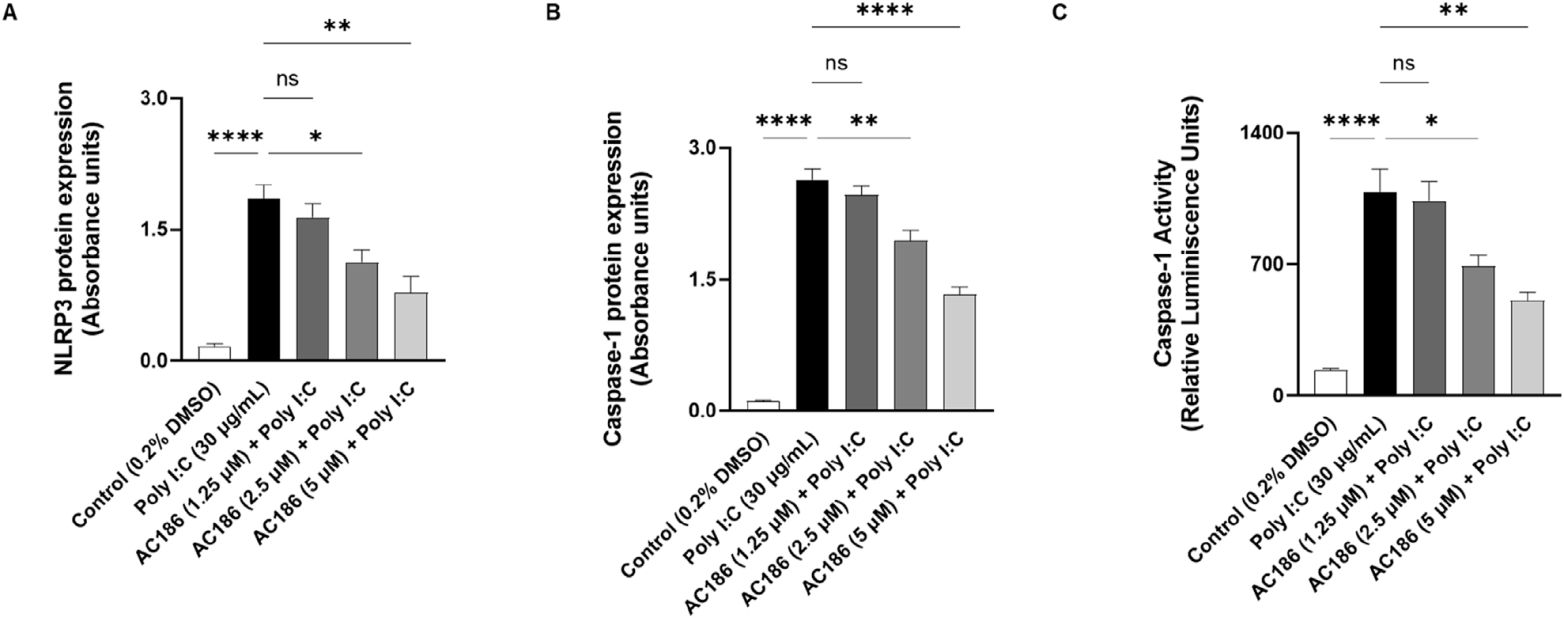
AC186 reduced poly I:C-induced activation of NLRP3/caspase-1 signalling. **(A)** In cell western ELISA experiments showing reduction in NLRP3 inflammasome protein expression in BEAS-2B cells pre-treated with AC186 (2.5 and 5 µM), prior to poly I:C stimulation. AC186 (2.5 and 5 µM) significantly (p < 0.05) inhibited poly I:C-induced increase in caspase-1 protein expression **(B)** and activity **(C)** in BEAS-2B cells stimulated with poly I:C (30 μg/mL). Results are presented as mean ± SEM for at least 3 independent experiments; *p < 0.05; **p < 0.01; ***p < 0.001; ****p < 0.0001; One way ANOVA with post-hoc Tukey’s test.

Poly I:C can activate toll-like receptor 3 (TLR3) to trigger the NLRP3 inflammasome, then caspase-1 and subsequently IL-1β. Based on the results showing that AC186 reduced elevated levels of IL-1β in BEAS-2B cells stimulated with poly I:C, experiments were conducted to evaluate effects of the compound on NLRP3/caspase-1 activation in the cells. Using in cell western ELISA, it was shown that poly I:C stimulation resulted in ∼8-fold increase in protein levels of NLRP3 inflammasome (Figure 5A). While AC186 (1.25 µM) did not produce significant (p < 0.05) reduction in poly I:C-induced increased NLRP3 protein expression, 2.5 and 5 µM of the compound produced ∼0.6 and ∼0.5 reduction (p < 0.05), respectively (Figure 5A). Results of further experiments showed that poly I:C stimulation of BEAS-2B cells caused significant (p < 0.0001) increases in both expression (Figure 5B) and activity (Figure 5C) of caspase-1. These increases were significantly (p < 0.05) reduced when cells were pre-treated with 2.5 and 5 μM, but not 1.25 µM concentration of the compound (Figures 5B,C).

### Anti-inflammatory activity of AC186 is dependent on oestrogen receptor

**FIGURE 6:**
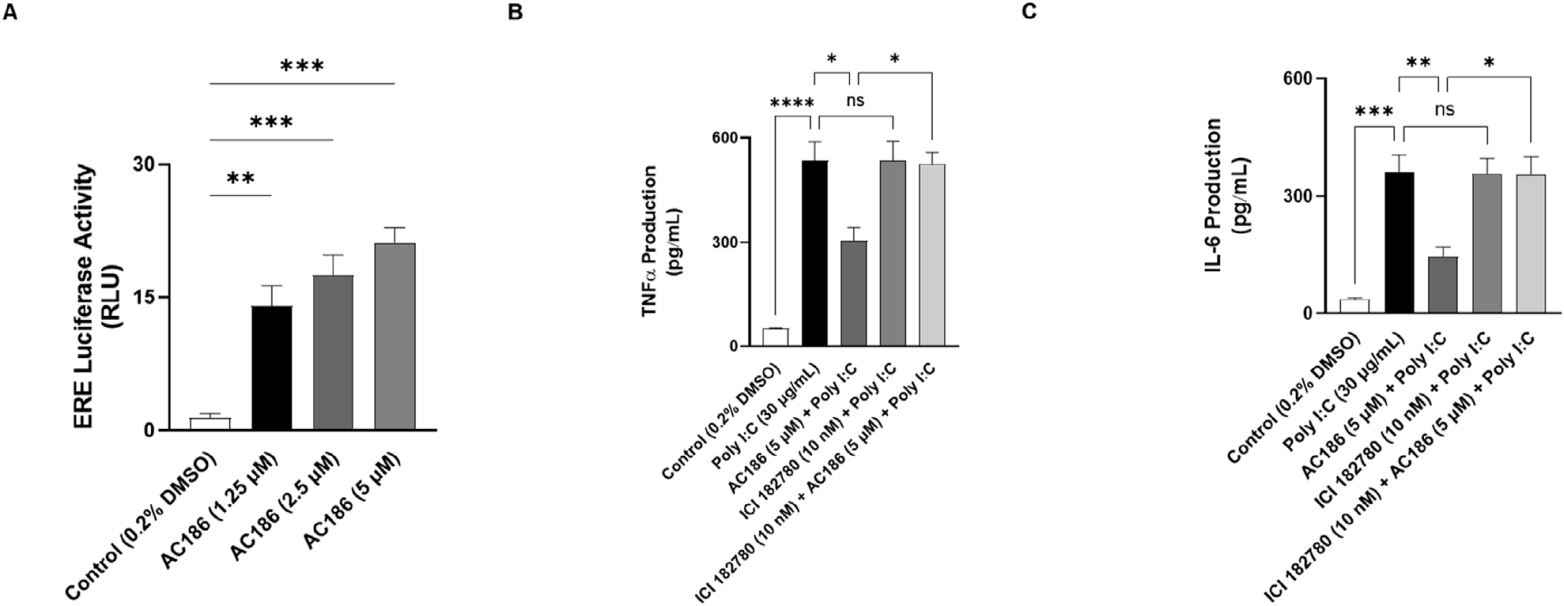
Anti-inflammatory activity of AC186 is dependent on oestrogen receptor. Treatment with AC186 (1.25, 2.5, and 5 µM) significantly (p < 0.05) increased ERE luciferase activity in BEAS-2B cells transfected with oestrogen receptor element (ERE) luciferase reporter vector **(A)**. Reduction in poly I:C-induced increased in TNFα **(B)** and IL-6 **(C)** production by AC186 (5 µM) was reversed in the presence of 182780 (10 nM). Results are presented as mean ± SEM for at least 3 independent experiments; *p < 0.05; **p < 0.01; ***p < 0.001; ****p < 0.0001; One way ANOVA with post-hoc Dunnett’s test.

AC186 is a known agonist at oestrogen receptors. This prompted experiments to determine whether this property of the compound may be contributing to its anti-inflammatory effects in BEAS-2B cells. To establish this, initial reporter gene assays were carried out to confirm effects of AC186 on the activity of oestrogen receptors in BEAS-2B cells. The outcome of these studies, shown in Figure 6A, revealed significant (p < 0.01) and concentration-dependent increase in ERE luciferase activity in BEAS-2B cells treated with AC186 (1.25, 2.5 and 5 µM).

This further prompted experiments to explore the impact of blocking oestrogen receptors on the anti-inflammatory effects of AC186 in BEAS-2B cells. BEAS-2B cells were treated with either AC186 (5 μM), or ICI 182780 (10 nM) + AC186 (5 μM) and then stimulated with poly I:C for 24 h. As shown in Figure 6B, ICI 182780, blocked the anti-inflammatory effect of AC186 on TNFα production in BEAS-2B cells. Similar results were obtained in experiments to determine the effects of ICI 182780 on the reduction of IL-6 by AC186 in poly I:C-stimulated BEAS-2B cells (Figure 6C).

### AC186 prevented poly I:C-induced bronchial epithelial cell death through mechanisms involving caspase-3/7

**FIGURE 7:**
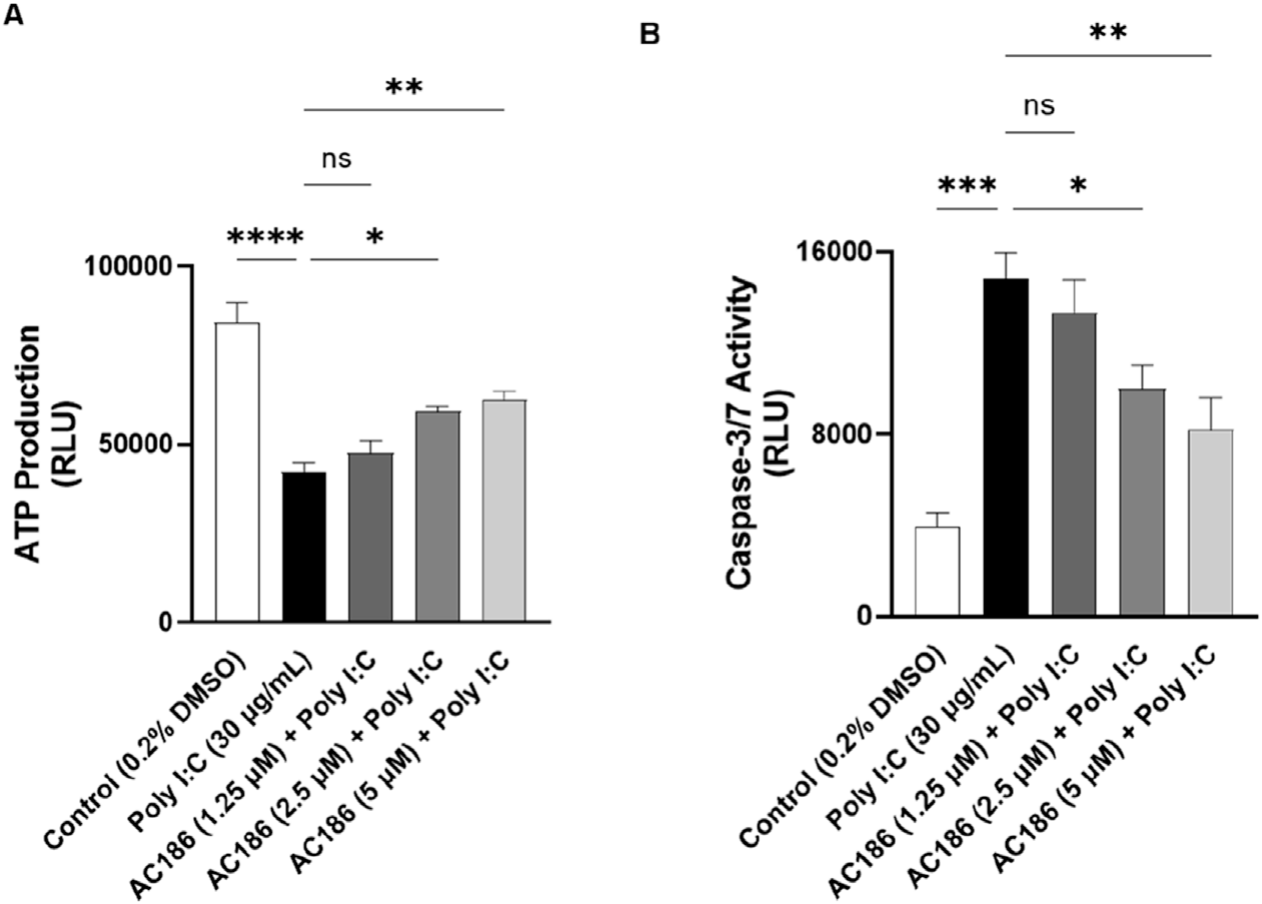
AC186 prevented poly I:C-induced BEAS-2B cell death through apoptotic mechanisms. Pre-treatment with AC186 (2.5 and 5 µM) significantly (p < 0.05) increased ATP production **(A)**, while reducing activities of caspase 3/7 **(B)** in BEAS-2B cells incubated with poly I:C (30 μg/mL) for 72 h. Results are presented as mean ± SEM for at least 3 independent experiments; *p < 0.05; **p < 0.01; ***p < 0.001; ****p < 0.0001; One way ANOVA with post-hoc Tukey’s test.

To evaluate the cytoprotective effect of AC186 against poly I:C-induced damage, BEAS-2B cells were treated with the compound, followed by incubation with poly I:C for 72 h. Viability assays showed that ATP production was significantly (p < 0.001) reduced in cells exposed to poly I:C alone, when compared with control cells (Figure 7A). However, pre-treatment with AC186 (1.25, 2.5 and 5 μ) resulted in significant (p < 0.05) improvement in viability, in comparison with poly I:C alone.

Furthermore, it was shown that incubating cells with poly I:C (30 μg/mL) resulted in significant elevation in caspase 3/7 activity, while pre-treatment with AC186 (5 and 10 μM) caused significant (p < 0.05) reduction in the activities of these apoptosis proteins (Figure 7B).

## Discussion

Most viral infections are closely linked to double-stranded RNA (dsRNA), which is either associated with viral genome or as a by-product during viral replication in the host (Chen and Hur, 2022). dsRNA acts as a pathogen associated molecular pattern (PAMP), which activates toll-like receptor-3 (TLR3) to trigger immune signalling pathways, resulting in the production of pro-inflammatory cytokines (Viana-de-Lima et al., 2025).

Polyinosinic: polycytidylic acid (poly I:C) is a synthetic dsRNA which mimics immune activation by viral infections. Poly I:C has been widely reported to activate TLR3 in human bronchial epithelial cells, resulting in inflammatory responses and impaired integrity of the airways (Royer et al., 2017; Shiratori et al., 2020; Sada et al., 2021). This study demonstrated that stimulation of human bronchial epithelial (BEAS-2B) cells with poly I:C for 24 h resulted in increased production of pro-inflammatory cytokines TNFα, IL-6 and IL-1β, as well as the chemokine IL-8. These experiments also showed that in the presence of the non-steroidal oestrogen receptor-beta (ERβ) agonist AC186, poly I:C-induced increased secretions of these mediators were markedly reduced. This is a significant outcome, considering the roles of these pro-inflammatory mediators in ARDS during infection by respiratory viruses. These results also reflect the outcome of a previous study demonstrating anti-inflammatory effects of AC186 in lipopolysaccharide-stimulated microglia (Katola et al., 2024).

In a systematic review and meta-analysis to establish an association between inflammatory biomarkers and ARDS risk, Liu et al. (2022) reported that ARDS is associated with a significantly elevated levels of IL-1β, IL-6, and TNFα. Similarly, the production of IL-1β, IL-6, IL-8, TNFα produced in human airway cells have been reported in patients with viral infection-induced respiratory diseases (Yamaya et al., 20203). The inhibitory effects of AC186 on the production of these mediators in response to poly I:C in bronchial epithelial cells therefore suggests potential value as a pharmacological intervention in viral infection-induced lung injury, with associated clinical benefits like improvement of symptoms.

Stimulation of BEAS-2B cells with poly I:C induce production of pro-inflammatory cytokines and chemokines through activation of the NF-κB transcription factor (Matsukura et al., 2006; Koarai et al., 2010; Bach et al., 2014). Consequently, experiments were carried out to determine whether inhibition of NF-κB activation contributed to the anti-inflammatory activity of AC186. The outcome of these experiments revealed that AC186 prevented cytoplasmic activation, DNA binding and subsequent transcriptional activity of NF-κB following stimulation of BEAS-2B cells. Similar to its effects on pro-inflammatory cytokines, earlier studies have shown that this compound caused a reduction in pro-inflammatory cytokines in LPS-stimulated microglia through mechanisms linked to NF-κB activation (Katola et al., 2024). Interestingly, LPS stimulation of microglia results in activation of (toll-like receptors 4) TLR4, to trigger NF-κB signalling (Zusso et al., 2019). It appears that AC186 can target NF-κB signalling to prevent the release of pro-inflammatory cytokines, following activation of either TLR3 or TLR4. This is, however, worthy of further investigation.

Studies have suggested that TLR3/NF-κB-mediated production of the pro-inflammatory cytokine IL-1β is achieved through activation of the NLRP3 inflammasome, and caspase-1 which cleaves pro-IL-1β to form the mature IL-1β (Grishman et al., 2012). Results of experiments from this study revealed reduction in poly I:C-induced NLRP3 and caspase-1 activation in BEAS-2B cells by AC186, suggesting that the NLRP3/caspase-1 pathway may be partially responsible for the effect of the compound on IL-1β secretion.

AC186 is a non-steroidal agonist at ERβ receptor (McFarland et al., 2013), and has been reported to produce ERβ-dependent anti-inflammatory activity (Katola et al., 2024). Results of experiments to determine possible involvement of ERβ in the anti-inflammatory activity of AC186 in poly I:C-stimulated BEAS-2B cells revealed an increase in ERE transcriptional activity, as well as partial reversal of anti-inflammatory activity in the presence of the steroidal oestrogen receptor antagonist ICI 182780. Oestrogenic compounds are known to produce anti-inflammatory activity (Vegeto et al., 2000; Cuzzocrea et al., 2000), through inhibition of NF-κB activation (Ghisletti et al., 2005; Mu et al., 2016; Li et al., 2024). In studies reported by Chotirmall et al. (2010), 17β-estradiol was shown to produce anti-inflammatory activity in bronchial epithelial cells stimulated with LPS. *In vivo*, this hormone reduced levels of pro-inflammatory cytokines in LPS-injured lungs of mice (Speyer et al., 2005). It is therefore proposed that the anti-inflammatory activity of AC186 may be linked to its oestrogen receptor agonist activity, and thus warrants further investigation.

Stimulation of bronchial epithelial cells with poly I:C has been reported to induce caspase-dependent apoptosis and death (Koizumi et al., 2016). In this study poly I:C induced inflammatory responses in BEAS-2B cells after 24 h while apoptosis and cell death were observed after 72 h. These are consistent with outcomes of similar studies reported previously; Ieki et al. (2004) were able to demonstrate an inflammatory response to poly I:C after 24 h, results published Koizumi et al. (2016) indicated that cell death and apoptosis became evident after 72 h. Interestingly, in addition to reducing inflammation induced after 24 h, AC186 also prevented poly I:C-induced apoptosis and damage to bronchial epithelial cells after 72 h.

The outcomes of this study suggest that AC-186 is an anti-inflammatory activity in poly I:C-stimulated BEAS-2 cells, through mechanisms involving inhibition of NF-κB, as well as the NLRP3/caspase-1/IL-1β activation pathways. Results also suggest that oestrogen agonist property may be contributing to its anti-inflammatory activity. It is proposed that AC186 may provide therapeutic benefits in reducing inflammatory events associated with ARDS caused by viral infections.

## Data Availability

Data can be obtained from the corresponding author upon reasonable request.
